# Prevalence, awareness and risk factors of hypertension in southwest of Iran

**DOI:** 10.12861/jrip.2015.11

**Published:** 2015-06-01

**Authors:** Leila Yazdanpanah, Hajieh Shahbazian, Heshmatollah Shahbazian, Seyed-Mahmuod Latifi

**Affiliations:** ^1^Health Research Institute, Diabetes Research Center, Ahvaz Jundishapur University of Medical Sciences, Ahvaz, Iran

**Keywords:** Hypertension, Blood pressure, Prevalence

## Abstract

**Introduction:** Hypertension is an important cause of stroke, heart and kidney disease and these diseases are the cause for about two-thirds of all mortalities around the world.

**Objectives:** The aim of this study was to assess the prevalence, awareness and risk factors of hypertension in Ahvaz, southwest of Iran.

**Patients and Methods:** In this descriptive-analytical study, 944 participants older than 20 years were enrolled. Systolic blood pressure (BP) ≥140 mm Hg, diastolic BP ≥90 mm Hg or the use of antihypertensive medication was considered as hypertension. Systolic BP = 140-159 mm Hg or diastolic BP = 90-99 mm Hg were defined as stage 1, and systolic BP ≥160 mm Hg or diastolic BP ≥100 mm Hg were considered as stage 2 of hypertension. Systolic BP = 120-139 mm Hg and diastolic BP= 80-89 mm Hg were considered as prehypertensive state.

**Results:** The prevalence of hypertension in Ahvaz was 17.58% (95% CI: 15.28-20.14) (males; 45.8%, females; 54.2%). Age-adjusted prevalence of hypertension was 8.6%; age- and sex-adjusted prevalence of hypertension was 3.7%. Seventy-two cases (7.7%) were prehypertensive. The frequency of stage 1 hypertension was 10.8% and stage 2 was 5.7%. Among them, 53.6% were not aware of their disease and 22% of hypertensive cases were controlled. Logistic regression analysis showed that age, metabolic syndrome and family history of hypertension had significant relationship with hypertension.

**Conclusion:** This study showed that, age, metabolic syndrome and family history of disease are risk factors of hypertension in Ahvaz population. About half of patients were unaware of their disease and about 20% had controlled BP.

**Implication for health policy/practice/research/medical education:**

This study showed that age, metabolic syndrome and family history of hypertension are risk factors of high blood pressure in Ahvaz population. About half of patients were unaware of their disease and about 20% had controlled blood pressure.

## Introduction

According to the last World Health Organization (WHO) report, noncommunicable disease prevalence is increasing. These diseases are the cause for about two-thirds of all mortalities around the world. According to this report; hypertension (the cause of half of deaths from stroke and heart disease) is present in one in 3 adults worldwide (1). Hypertension is a risk factor for kidney disease (2) and the most important preventable risk factor for cardiovascular disease (3).

The prevalence of hypertension increases with age. Systolic blood pressure (BP) has a progressive rise during lifetime with a difference of 20-30 mm Hg between early and late adulthood. Diastolic BP tends to be consistent until the fifth decade. The mean systolic and diastolic BP in men is higher than women in early adulthood, but this difference reverses by the sixth or seventh decade. In fact, the risk of developing hypertension in healthy people after the sixth decade is about 90% (3).

The worldwide prevalence of hypertension varies from place to place. The lowest prevalence was reported in rural India (3.4% in men and 6.8% in women) and the highest in Poland (68.9% in men and 72.5% women). Awareness of the disease, varied from 25.2% in Korea to 75% in Barbados. Controlled BP after treatment varied from 5.4% in Korea to 58% in Barbados (4).

Studies in Iran have reported different results. In the last national study in 2005, 25.2% (6.6 million cases) of Iranian people aged 25-64 years had high BP, 45.5% were prehypertensive, 34% were aware of their disease and 25% were taking medication to lower BP however only 24%were controlled (2).

Risk factors for high BP consist of 2 categories: nonmodifiable risk factors including age, gender, race, genetic factors, (5) and modifiable factors such as physical inactivity, obesity and high intake of calories, high levels of dietary sodium intake and alcohol consumption. Daily sodium intake and obesity are the 2 most important risk factors because they have a direct relationship with kidney disease (6).

High BP may have no warning signs and it can be diagnosed only by BP measurement. This is the reason that screening is the best strategy to find the patients. Studies of hypertension in developing and developed countries did not show significant differences in mean prevalence, awareness, treatment and control of hypertension (7). In recent reports in high-income countries, diagnosis and treatment have reduced mean BP in the population. In developing countries, most people remain undiagnosed, although many of them could be treated at a low expense (1).

## Objectives

Prevalence of high BP differs worldwide and hypertension prevalence depends on the diagnostic criteria, methods and studied population. This study was done to determine the prevalence, awareness and risk factors of hypertension in Ahvaz, south west of Iran.

## Patients and Methods

### Study patients

This descriptive-analytical study was performed with random cluster sampling method in population older than 20 years in Ahvaz, southwest of Iran in 944 participants. The clusters were 6 public health centers randomly selected from 24 centers. The procedure was described for patients and written consent was completed by all of them.

A checklist including age, sex, BP, marital status, educational level, ethnicity, body mass index (BMI), waist circumference, high BP history, history of diabetes, antihypertensive drug consumption and family history of hypertension was completed for all participants.

### Laboratory measurements

After 12 hours of fasting, blood samples were taken in the morning. Then fasting blood sugar (FBS), serum triglyceride (TG), cholesterol (Chol) and HDL-C were measured using an enzymatic colorimetric method with Pars Azmoon kit. (With Biotechnical instruments model BT-3000 Germany).

### Blood pressure measurement

BP was measured by standard sphygmomanometer (Yamasu Desk Models UN600) and sized cuff after 15 minutes rest in sitting position. The cuff was fixed on right arm that was supported at heart level. Then the cuff was inflated 30 mm Hg above radial pulse obliteration level. Systolic BP was considered as the point at which the first Korotkoff sound was heard, and disappearance of Korotkoff sound was defined as diastolic BP. Two measurements were obtained at 15 minutes intervals and their average were recorded as patients’ BP. We assured that caffeine, exercise, smoking and some drug consumption was avoided for at least 30 minutes prior to measurement (8).

According to the eighth report of the joint national committee on prevention, detection, evaluation, and treatment of high BP (JNC8), systolic BP ≥140 mm Hg or diastolic BP ≥90 mm Hg or use of antihypertensive medication, was considered as hypertension. Systolic BP = 140-159 mm Hg or diastolic BP =  90-99 mm Hg was defined as stage 1 of hypertension, systolic BP ≥160 mm Hg or diastolic BP ≥100 mm Hg was considered as stage 2 of hypertension. Those who were not hypertensive but had systolic BP = 120-139 mm Hg and diastolic BP = 89-80 mm Hg were considered as prehypertensive. Systolic BP <140 mm Hg and diastolic BP <90 mm Hg in patients taking medication was defined as controlled hypertension (9).

### Anthropometric measurements

Weight and height were measured without shoes with light clothing. Waist circumference was considered as midline of the lower ribs and upper outer edge of the right iliac crest. Abdominal obesity was defined as waist circumference greater than 102 cm in men and 88 cm in women (8). Waist-to-hip ratio is an indicator of body fat distribution. According to National Institute of Diabetes and Digestive and Kidney Diseases (NIDDK) classification, waist-to-hip ratio less than 0.95 in men and less than 0.8 in women is considered normal (10).

### Diagnosis of metabolic syndrome

For diagnosis of metabolic syndrome at least 3 of the following 5 components were considered necessary (according to ATP III criteria update 2005) (8).

1- Abdominal obesity (waist circumference ≥102 cm in men and ≥88 cm in women)
2- TG ≥150 mg/dl or history of drug taking for hypertriglyceridemia.
3-HDL-C ≤40 mg/dl in men and ≤50 mg/dl in women or history of drug taking
4- BP ≥130/85 mm/Hg or history of hypertensive drug consumption
5- FBS ≥100 mg/dl, history of diabetes mellitus or use of antidiabetic drugs.

Diabetes was considered as fasting glucose ≥126 mg/dl or taking hypoglycemic medications. Hypercholesterolemia was defined as serum cholesterol ≥200 mg/dl. Education level was defined as illiterate, primary school, diploma, and above diploma. Awareness was defined as previous history of hypertension.

### Ethical issues

1) The research followed the tenets of the Declaration of Helsinki; 2) informed consent was obtained; and 3) This study was approved by the Ethics Committee of Ahvaz Jundishapur University of Medical Sciences.

### Statistical analysis

Descriptive statistics were used to present tables and figures. Chi-square test, independent *t* test and logistic regression models were used for communication survey. In these models, the independent variables were age, sex, family history, ethnicity, marital status, educational level, waist circumference, FBS, metabolic syndrome and the dependent variable was hypertension. Hypertension variable was defined as a binary variable. Maximum likelihood method was used to estimate the model efficiency. Educational level was defined as illiterate, primary school, diploma, above diploma and illiterate was the reference in model. In marital status single was reference, for sex, male was reference, for ethnicity non-Arab was reference. Data were analyzed by SPSS version 19 software.

In this study, *P* ≤ 0.05 was considered as significant. The 2011 Iran National Census Report (11) was used to evaluate the age and sex adjusted prevalence.

## Results

Prevalence of hypertension in Ahvaz population was 17.58 (95% CI: 15.28-20.14). Among 166 hypertensive cases, 76 (45.8%) of them were male and 90 (54.2%) were female. Age-adjusted prevalence of hypertension was 8.6%; age- and sex-adjusted prevalence was 3.7%. Table 1  shows the characteristics of study participants.

**Table 1:** Characteristics of participants

	**Normal** **(n=700)** **(Mean ± SD)**	**Prehypertension** **(n=72)** **(Mean ± SD)**	**Hypertension** **(n=72)** **(Mean ± SD)**
Age (years)	38.5 ± 12.5	48.1 ± 12.0	55.4 ± 11.7
BMI (kg/m^2^)	26.5 ± 4.7	27.8 ± 4.1	28.8 ± 4.4
Waist (cm)			
Men	89 ± 10.6	91.1 ± 10.6	95.5 ± 9.9
Women	81.1 ± 11	87.7 ± 12.4	92.55 ± 11.2
Systolic BP (mm Hg)	107.6 ± 16.9	129.6 ± 2.3	140.4 ± 18.2
Diastolic BP (mm Hg)	67 ± 13.7	75.7 ± 7.4	87.3 ± 12.8
Cholesterol (mg/dl)	200.9 ± 44.6	207.9 ± 42.1	221.4 ± 46
FBS (mg/dl)	101.9 ± 38.2	111 ± 47.2	122.4 ± 56.8

Abbreviations: BMI, body mass index; BP, blood pressure; FBS, fasting blood sugar.

Seventy-two cases (7.7%) were prehypertensive. The frequency of stage 1 hypertension was 102 patients (10.8%) and stage 2 was 54 cases (5.7%). Forty-six patients (27.7%) were taking antihypertensive medication. Frequency of BP in participants with different age and sex groups is shown in  Table 2.

**Table 2:** Frequency of different blood pressure groups in participants with different sex and age groups

	**Normotension** **No. (%)**	**Prehypertension** **No. (%)**	**Hypertension**	
			**Stage 1 No. (%)**	**Stage 2 No. (%)**
Male				
20-29	84 (26.2)	4 (9.5)	0 (0)	0 (0)
30-39	67 (20.9)	6 (14.3)	7 (13.5)	1 (5.3)
40-49	87 (27.1)	12 (28.6)	8 (15.4)	4 (21.1)
50-59	58 (18.1)	12 (28.6)	16 (30.8)	7 (36.8)
60-69	21 (6.5)	7 (16.7)	8 (15.4)	4 (21.1)
70‏≥	4 (1.2)	1 (2.4)	13 (25)	3 (15.8)
Female				
20-29	117 (32.1)	0 (0)	1 (2)	1 (2.9)
30-39	106 (29)	7 (23.3)	3 (6)	5 (14.3)
40-49	88 (24.1)	9 (30)	10 (20)	5 (14.3)
50-59	39 (10.7)	8 (26.7)	21 (42)	11 (31.4)
60-69	12 (3.3)	5 (16.7)	12 (24)	10 (28.6)
70≥	3 (0.8)	1 (3.3)	3 (6)	3 (8.6)
Total				
20-29	201 (29.3)	4 (5.6)	1 (1)	1 (1.9)
30-39	173 (25.2)	13 (18.1)	10 (9.8)	6 (11.1)
40-49	175 (25.5)	21 (29.2)	18 (17.6)	9 (16.7)
50-59	97 (14.1)	20 (27.8)	37 (36.3)	18 (33.3)
60-69	33 (4.8)	12 (16.7)	20 (19.6)	14 (25.9)
70‏≥	7 (1)	2 (2.8)	16 (15.7)	6 (11.1)

Among hypertensive patients, 89 cases (53.6%) were not aware of their disease and 77 cases (46.4%) were aware. Twenty-two percent of all hypertensive cases were controlled (48% of aware participants were controlled). Fifty percent of women and 42.1% of men were aware of their disease. There was no statistically significant difference in awareness between the two genders (*P* = 0.31) (  Table 3). In patients without metabolic syndrome, 62 patients (8.8%) and in patients with metabolic syndrome, 100 (48.1%) were hypertensive (*P* = 0.0001).

**Table 3:** Participants with hypertension that are aware and controlled by sex and age

**Age (year)**	**Hypertensive men (%)**		**Hypertensive women (%)**		**Total (%) **	
	**Aware **	**Controlled**	**Aware**	**Controlled**	**Aware **	**Controlled**
20-39	3.1	0	4.4	4.4	3.9	2.6
40-49	21.9	15.6	20	11.1	20.8	13
50-59	34.4	18.8	46.7	17.8	41.6	18.2
60-69	21.9	15.6	22.2	6.7	22.1	10.4
70‏ ≥	18.8	3.1	6.7	4.4	11.7	3.9

Of all participants 14.1% were single and 85.9% married. Thirty-nine percent (19.1% of men and 56.8% of women) had high waist-to-hip ratio. In total, 14.2% of participants with Arab ethnicity and 21.4% of participants with non-Arab ethnicity had hypertension.

Along with increasing age, the risk of hypertension increased from 0.5% in the 20-29 years age group to 70% in the above 70 years age group, which showed a statistically significant correlation (*P* = 0.0001).

Prevalence of hypertension was 23.6% in cases with family history of hypertension and 13.9% in those without family history. The difference between the 2 groups was significant (*P* = 0.0001).

A logistic regression analysis was made to assess the influence of various factors on hypertension. The risk of hypertension increased with age; from . Odds ratio (OR)= 1 in 20-29 years group to OR=443.6 in ≥70 years group. OR in participants with family history of hypertension was nearly twice compared to those without family history. OR in cases with metabolic syndrome was nearly 10 times more compared to cases without metabolic syndrome. Other factors (sex, marital status, educational level, waist circumference, BMI, FBS, ethnicity) did not show significant correlation with hypertension (  Table 4).

**Table 4:** Logistic regression model results

	*** P *** **-value**	**OR**	**CI**	
			**Lower**	**Upper**
Sex (ref: male)	0.677	0.879	0.478	1.616
Age (ref: 20-29)	0.000			
30-39	0.019	13.706	1.526	123.118
40-49	0.007	20.642	2.283	186.657
50-59	0.000	56.559	6.326	505.649
60-69	0.000	88.746	9.392	838.573
≥70	0.000	421.072	38.781	4571.913
Family history	0.010	1.895	1.166	3.079
Ethnicity (ref: non-Arab)	0.121	1.550	0.891	2.695
Marital status	0.342	0.534	0.146	1.949
Educational level (ref: illiterate)	0.494	0.827	0.480	1.426
Waist circumference	0.564	1.012	0.973	1.052
FBS	0.762	0.999	0.994	1.004
Metabolic syndrome	0.000	9.260	5.208	16.465

Abbreviations: FBS, fasting blood sugar; OR, odds ratio.

**Figure 1:**
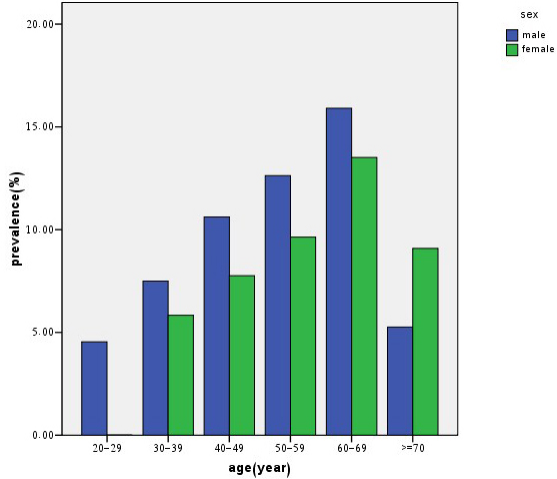
Prevalence of hypertension by age and sex.

**Figure 2:**
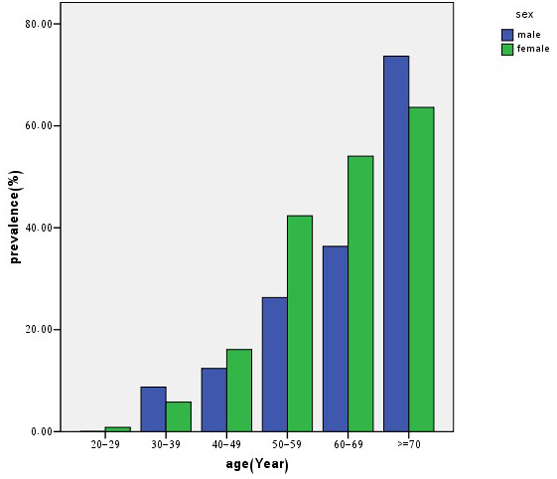
Prevalence of prehypertension by age and sex.

Prevalence of hypertension and prehypertension by age and sex is shown in Figures 1 and 2, respectively.

## Discussion

The aim of this study was to evaluate prevalence, awareness and risk factors of hypertension in Ahvaz, Iran. We found that prevalence of hypertension in Ahvaz population was 17.58%. Among 166 hypertensive cases, 76 (45.8%) of them were male and 90 (54.2%) were female. Age-adjusted prevalence of hypertension was 8.6%; age- and sex-adjusted prevalence was 3.7%. Compared to other studies, this was not a high prevalence. Hypertension prevalence was lower than the United States, European countries reports and also some developing countries (12-17). In previous Iranian studies, the prevalence of hypertension was reported as 13.9%-42.7% (2,18-23,24). In our study, the hypertension prevalence was near the lower extreme of these studies, however the difference of participants ages, time of the study and ethnicity might explain these differences.

The prevalence of stage 1 hypertension was 10.8% and stage 2 was 5.7% in this study, which was lower than similar studies (2,20).

The prevalence of prehypertension was 7.7% in our study. It was lower than similar studies (2,22), however the difference of age and sex between study groups may be the cause of this difference.

In this study 46.4% were aware of their hypertension, which was comparable with some other studies (2,13,14,16,20,24) and was lower than conducted studies in the United States (17), however it was higher than Kenya and Tanzania (15,25). This may be due to educational and economic status.

We found that 22% of patients had controlled BP, that was comparable with most of the worldwide studies (13,15,20,24,25), but it was lower than the United States (17). Economic status may explain the lower prevalence compared to developed countries. It was also higher than a previous study in Iran (2) and some other studies (14,16). These differences may be due to screening, education level and socioeconomic status.

After logistic regression analysis to evaluate influence of some risk factors on hypertension, we found that age, metabolic syndrome and family history of hypertension had significant correlation with hypertension. In most of the other studies, age showed a relationship with hypertension (15,18,22,26,27). Mancia et al (28) found a correlation between metabolic syndrome and hypertension. In the studies by Kaur et al (26) and Ahmadi et al (27), family history of hypertension was related to the presence of hypertension which was in accordance to our study.

In other studies BMI (14,17,21-27), FBS (27), sex and ethnicity (23) were related to hypertension, In this study, however, these factors did not show any significant correlation using regression analysis. Logistic regression showed significant correlation between hypertension and BMI when metabolic syndrome was not considered as a variable in the model, but it did not show significant correlation when metabolic syndrome was considered. It seems that metabolic syndrome variable covers the BMI variable effect on hypertension in the model. The differences between risk factors may be a result of differences in age, ethnicity and lifestyle in study groups.

According to this study due to a high prevalence of hypertension and unawareness of disease, screening and education of general population to diagnose and control this disease is important to prevent sever and debilitating complications of hypertension. Health policymakers can use these data to design better strategies to improve awareness and control of hypertension.

## Conclusion

This study showed that age, metabolic syndrome and family history of disease are the risk factors of hypertension in Ahvaz population. About half of patients were unaware of their disease and only about one-fifth of them had controlled BP. Therefore health promotion programs are necessary to improve hypertension diagnosis and management.

## Limitations of the study

It was better to define hypertensive patients by holter monitoring of BP. Also it was better to examine the BP more than 2 times or in different days, however, we examined 2 times just in the same day.

## Acknowledgments

Special thanks to Dr Hamed Tabesh for statistical consult and Mrs Caroline Kheradmand for editing and thanks to Ahvaz Golestan Hospital Clinical Development Research Unit.

## Authors’ contribution

HBSH and HSH designed the research, HBSH and LY conducted the research, SL analyzed the data, and LY prepared the primary draft. LY, HBSH, HSH and SL critically reviewed and gave the final approval. All authors contributed equally to data acquisition.

## Conflicts of interest

None.

## Funding/Support

This paper is issued from research project (D-8701). Financial support was provided by Diabetes Research Center, Ahvaz Jundishapur University of Medical Sciences.
